# A Novel Targeted Analysis of Peripheral Steroids by Ultra-Performance Supercritical Fluid Chromatography Hyphenated to Tandem Mass Spectrometry

**DOI:** 10.1038/s41598-018-35007-0

**Published:** 2018-11-19

**Authors:** Neil de Kock, Santosh R. Acharya, S. J. Kumari A. Ubhayasekera, Jonas Bergquist

**Affiliations:** Department of Chemistry – Biomedical Center, Analytical Chemistry, Uppsala University, Box 599, Uppsala, 75124 Sweden

## Abstract

Ultra-performance supercritical fluid chromatography–tandem mass spectrometry (UPSFC–MS/MS) is an alternative method for steroid analysis. Continuous development of analytical methodologies for steroid profiling is of major importance in the clinical environment to provide useful and more comprehensive data. The aim of this study was to identify and quantify a large number of endogenous steroids from the four major classes (estrogens, androgens, progestogens and corticosteroids) simultaneously within a short analytical time. This novel UPSFC–MS/MS method with electrospray in positive ionisation (ESI+) mode is robust, selective and present sufficiently high sensitivity to profile nineteen steroids in 50 µL human plasma. Under optimised conditions, nineteen different steroids were separated with high efficiency in the multiple reaction monitoring (MRM) mode. The linearity of the method was good with correlation coefficients (R^2^) in the range of 0.9983–0.9999 and with calibration range from 0.05–500 ng/mL in human plasma. The intraday and interday precision of the method, as RSD, was less than 15%. The accuracy of the nineteen analytes varied between 80 to 116%. Finally, the novel method was successfully applied for the determination of nineteen steroids within 5 minutes providing the possibility to use it for research as well as routine healthcare practice.

## Introduction

**Figure 1:**
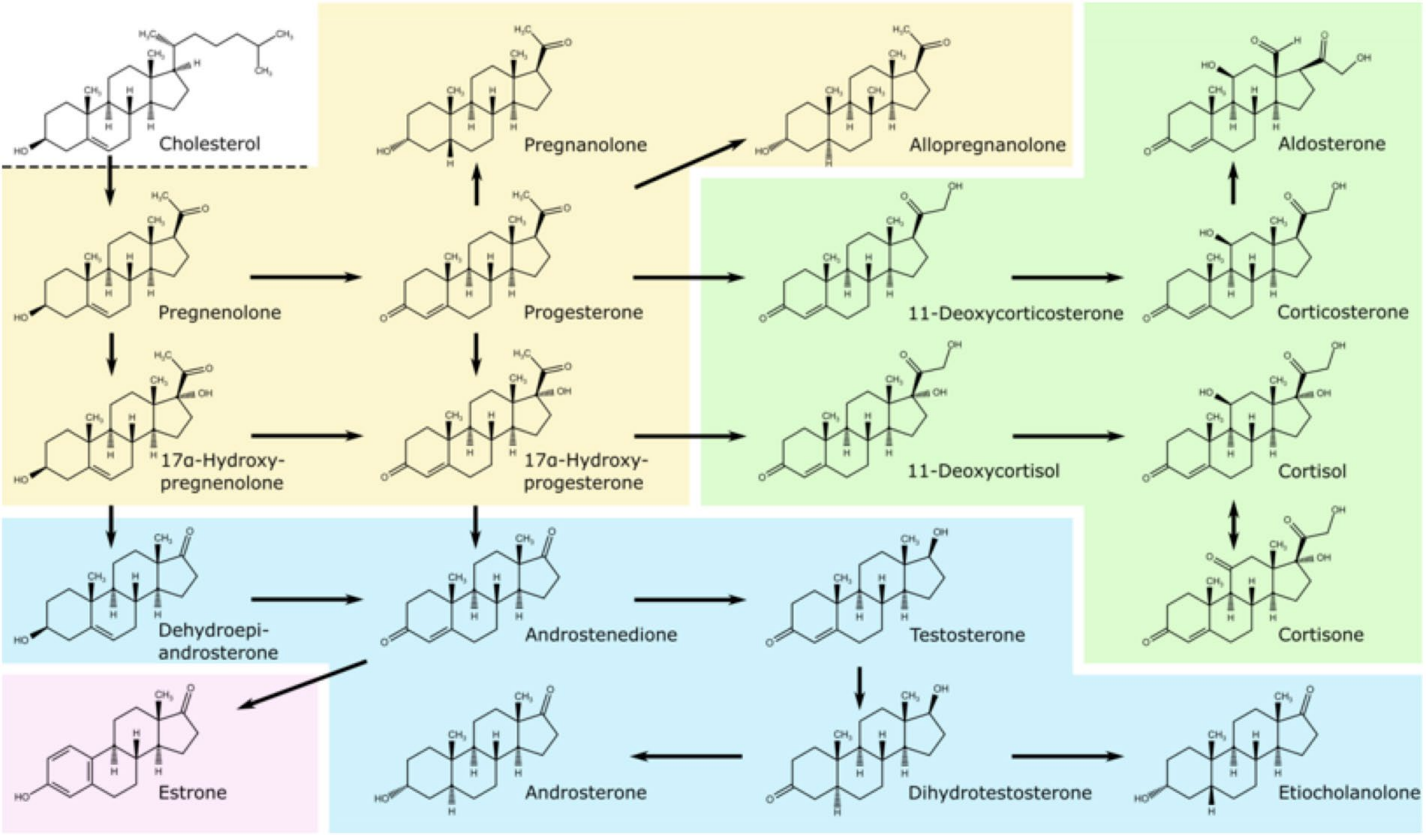
Steroid hormone biosynthesis pathway with some steroid metabolites in the human body. The four steroid classes are progestogens (yellow), corticosteroids (green), androgens (blue) and estrogens (pink).

Endogenous steroids such as estrogens, androgens, progestogens, corticosteroids, and their metabolites are naturally occurring physiologically important compounds controlling different functions in the human body^1^. These compounds derive from cholesterol, which is predominantly synthesised *de novo* in all aminals including human cells^2^. Steroids are formed during steroidogenesis of cholesterol (Fig. 1) in many tissues, including the brain, adrenal glands, gonads, and placenta^3^.

During the last two decades, there has been an increased focus on the application of steroids as possible biomarkers in healthcare practice. Depletion of steroid hormones with age is a well-known fact and has been implicated in some endocrine and metabolic diseases^4–10^.

The analytical methodologies based on chromatography and tandem mass spectrometryfor the determination of steroids in biological samples have  obtained profound consideration in recent past. Steroid profiling in routine clinical diagnosis is an essential source of information on different disorders^4–7,10^. Therefore, an accurate analysis of steroids in biological tissues has become important for contemporary medicine, even if troublesome, especially due to the minute concentration levels in certain biological samples^6^.

Several techniques are used for the quantification of steroids. The most common methods for steroid quantification in clinical practice include immunoassays, i.e. radioimmunoassay or enzyme immunoassay. The main disadvantages of immunoassay techniques are the cross reactivity of the antibodies used in the assay with the related steroids, and being prone to matrix effects^6,7^. In recent past, most of the separation methods of two or more steroids are based on either liquid chromatography (LC) or gas chromatography (GC) coupled to tandem mass spectrometry (MS/MS). These methods offer simultaneous determination of steroids from the four major classes (estrogens, androgens, progestogens and corticosteroids), and provide useful data in the clinical environment^7^. Moreover, these high-tech methods offer tremendous value in obtaining useful structural information on individual steroids and their metabolites^5^.

Analysis of steroids and their metabolites in biological samples with GC–MS is typically accompanied by different chemical derivatisation methods^11^. With the recent developments in MS, GC has been hyphenated with many different types of mass spectrometers, including triple quadrupole (TQ, tandem MS)^12^, in order to improve the sensitivity of the steroid analysis. Likewise, LC has been coupled to different MS systems with electrospray ionisation (ESI) and atmospheric pressure chemical ionisation (APCI) as the most common ionisation techniques^7^. Analysis of steroids without derivatisation by LC–MS/MS is well documented and is also widely used in the clinical practice^6,7^. The advantages of LC–MS/MS are less sample preparation and shorter analytical time in comparison to GC–MS/MS, with the latter providing better chromatographic resolution^5^.

Supercritical fluid chromatography (SFC) is an important analytical technique used for highly efficient separation with short analytical durations. Recent developments in SFC make it a powerful technique for the analysis of a wide range of compounds, including non-polar, polar, and ionisable analytes. SFC can exhibit different chromatographic behaviours such as normal-phase, reversed-phase, ion pairing or a combination of these dissimilar modes^13^. Fast and high resolution separations are achievable at reasonable pressures due to the lower viscosity of its main mobile phase (CO_2_). The key factors for SFC method development are a stationary phase to ensure good resolution and the addition of an appropriate co-solvent for analyte solvation. Moreover, SFC improves the separation of isomers and enantiomers compared to other separation techniques. Thus coupling of SFC with MS/MS provides several advantages related to sensitivity and specificity^14,15^. To the best of our knowledge there is so far not an UPSFC–MS/MS (UP denotes ultra-performance) method available for the simultaneous quantification of endogenous steroids from the four major classes in small volumes of human plasma. Our method allows for the determination of nineteen endogenous steroid hormone levels in 50 µL plasma, within a few minutes.

## Results and Discussion

**Figure 2:**
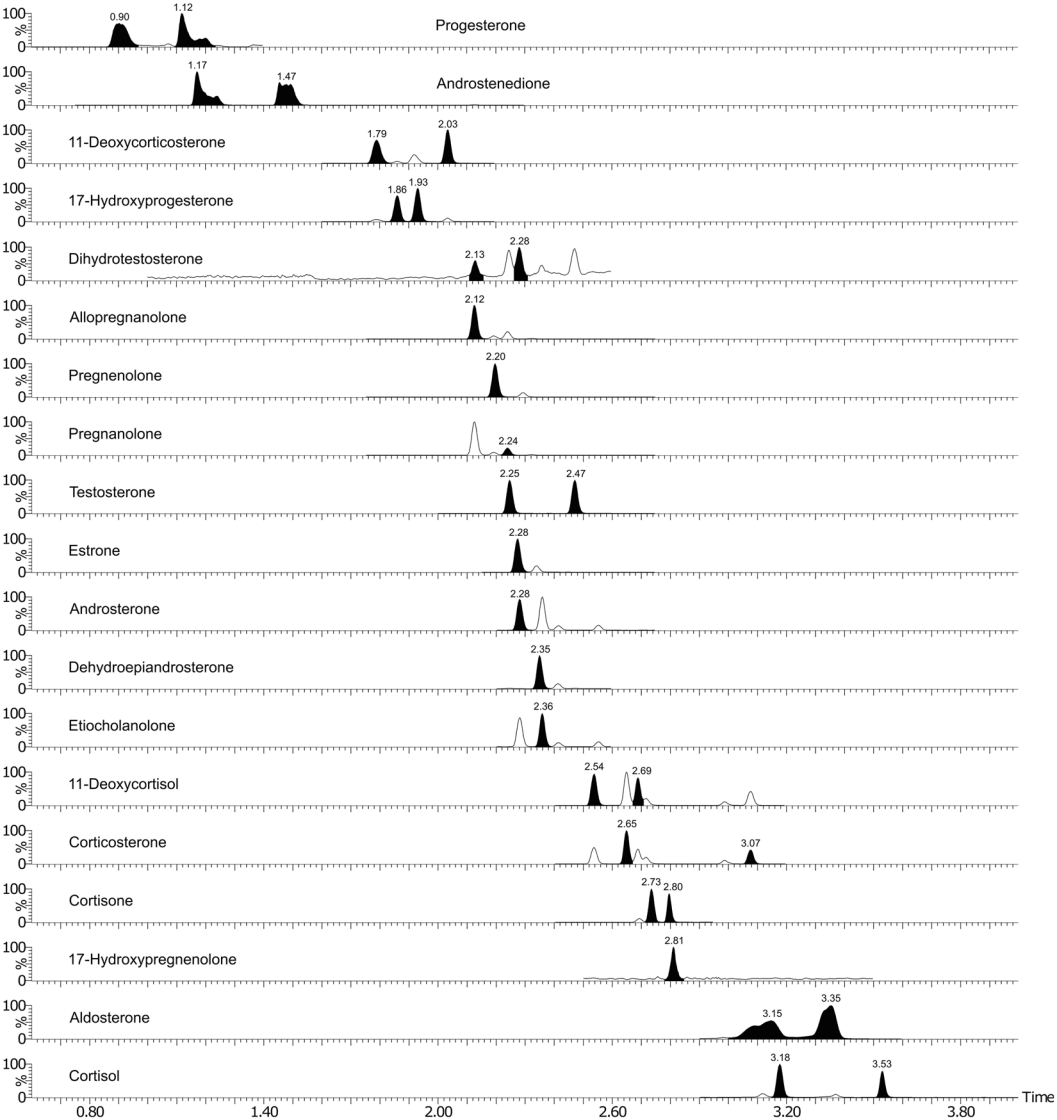
Typical chromatogram of representative steroids obtained in a single injection of spiked human plasma extract after derivatisation.

Separation of nineteen different endogenous steroid hormones and metabolites was successfully achieved within a 5 min run time using an UPSFC–MS/MS method (Fig. 2). The novelty of the present study is a fast, sensitive and reliable method for simultaneous quantification of endogenous steroids across the four major steroid classes. Most techniques reported for the analysis of steroids are focused on the determination of only a few steroids within one or two classes. It was reported in a recent review that authors of only 12.5% of all published reports, mentioned simultaneous analysis of 8 to 35 steroid analytes from all four major classes by using GC–MS/MS or LC–MS/MS methods^7^.

To the best of our knowledge there are very few reported studies of analysis of steroids and their metabolites using SFC–MS/MS^16–19^. Xu *et al*. analysed standards of the estrogenic class and its metabolites with a separation time of 10 min^16^. These steroids were derivatised with dansyl chloride prior to analysis. The chromatography setup consisted of two columns, a cyano-propyl silica column that was connected in series with a diol column for two dimensional analyses. This method was implemented to analyse only for estrogen metabolites. In our method estrone (E1) elutes at 2.17 min whereas with the other method E1 eluted at 5.25 min^16^. In a more recent publication, Quanson *et al*. described the development of a high-throughput analysis of underivatised androgenic steroids using a BEH 2-EP column^17^, and which was subsequently applied in a study by du Toit *et al*. to analyse eleven different oxygenated steroids in 4 min^20^. A new SFC–MS/MS method was also reported by Doué *et al*. for the analysis of eight glucuronide and ten sulphate steroids from the estrogenic and androgenic classes in urine. Glucuronide and sulphate steroids were fractioned and separated on a BEH column and a BEH 2-EP column, respectively. Each separation was accomplished within 8 min^19^. In the most recent publication, Parr *et al*. reported the analysis of 32 underivatised steroids with an analysis time of 21 min and LODs ranging from 1 to 50 ng/mL^18^. In comparison, our method is fast (5 min) with LODs less than 0.05 ng/mL for most of the steroids that we measured.

**Figure 3:**
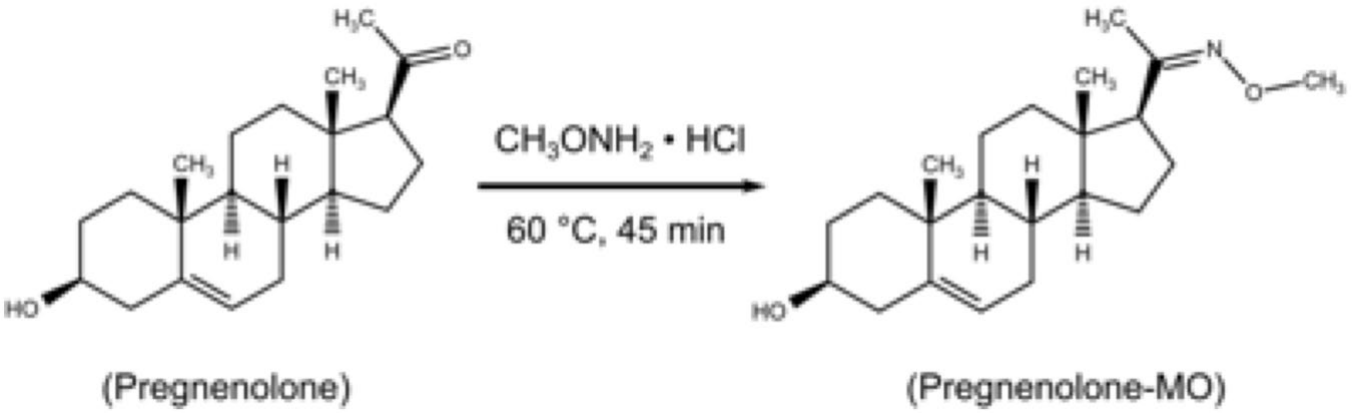
Chemical reaction of a steroid with methoxyamine (e.g. pregnenolone).

UPSFC has been connected to ESI, APCI and atmospheric pressure photoionisation (APPI) as ionisation sources for MS detection^15,18^. Parr *et al*. reported ESI+ to be superior for a steroid mixture^18^. Yet, the application of ESI–MS in steroid analysis is limited due to the lack of easily ionisable moieties in the steroid molecule. More explicitly, the carbonyl and hydroxyl groups are low proton affinity functional groups in the steroid molecules. Chemical addition to these functional groups in the steroid ring is needed to increase the ionisation capacity by protonation or deprotonation of the steroid molecule, which dramatically improves the ionisation efficiency (IE) of the analytes. There are several ways of derivatisation to increase IE of steroid analytes^11^. Here, we used methoxyamine (MO) which reacts with carbonyl groups to form the corresponding oximes (Fig. 3). The resulted oxime derivatives have improved IE and detectability of steroid analytes due to the higher proton affinity of the nitrogen-containing moiety. The fragmentation patterns increase sensitivity and selectivity, thus improving the detection of steroids. Furthermore, the derivatisation resulted in the formation of two isomers for eleven of the steroids (androstenedione, testosterone, dihydrotestosterone, progesterone, 17α-hydroxyprogesterone, cortisone, cortisol, corticosterone, 11-deoxycortisol, 11-deoxycorticosterone, and aldosterone) and both peaks were used during quantification of these eleven steroids. The 11-keto group did not react under the derivatisation conditions probably due to steric hindrance^11^. The corresponding peaks of geometric syn- and anti-isomers of oximes show baseline separation (Fig. 2). Furthermore, before reporting the data, we have established the optimum incubation condition of the MO derivatisation to be 45 min at 60 °C.

Mass spectrometric conditions were optimised using IntelliStart™ in infusion mode. The best results were obtained using ESI in positive mode for all nineteen steroids. Methanol with the addition of 0.1% formic acid as make-up solvent enhanced the IE especially for the compounds eluting at the beginning of the analysis probably due to the formation of a stable spray^19^. The [M + H]^+^ ion was selected as the precursor ion for each analyte and the highest intensity product ion(s) were selected to construct the MRM method. The collision energies for the MRM transitions were optimised for each steroid analyte and are reported in Table 1.

**Table 1:** Mass spectrometric parameters for the identification and quantification of the methoxime derivatives of steroids.

Class of analytes	Compound	Derivative	Corresponding internal standard	T_r_^a^ (min)	Precursor ion (*m/z*)	Product ions (*m/z*) (1/2/3)^b^	Collision energy (eV) (1/2/3)^c^	*D*_*t*_ (s)
Estrogens	Estrone	E1-MO	d_4_-E1-MO	2.28	300.1	157.0/253.2	25/15	0.004
Androgens	Dehydroepiandrosterone	DHEA-MO	d_2_-AN-MO	2.35	318.3	110.2/253.2/286.2	25/17/18	0.004
	Androsterone	AN-MO	d_2_-AN-MO	2.28	320.3	255.1/288.2	18/18	0.004
	Etiocholanolone	ECN-MO	d_2_-AN-MO	2.36	320.1	255.1/288.2	18/18	0.004
	Androstenedione	AE-diMO	d_9_-P-diMO	1.17/1.47	345.3	260.2/283.2	27/26	0.019
	Testosterone	T-MO	^13^C_3_-T-MO	2.25/2.47	318.1	126.1/138.0	29/30	0.004
	Dihydrotestosterone	DHT-MO	^13^C_3_-T-MO	2.13/2.28	320.2	128.3/140.2	29/30	0.004
Progestogens	Pregnenolone	Preg-MO	^13^C_2_-d_2_-Preg-MO	2.20	346.2	100.1/300.1	23/26	0.004
	17α-Hydroxypregnenolone	17OHPreg-MO	^13^C_2_-d_2_-Preg-MO	2.81	362.3	344.1	5	0.033
	Progesterone	P-diMO	d_9_-P-diMO	0.90/1.12	373.1	286.2/327.2	28/28	0.032
	17α-Hydroxyprogesterone	17OHP-diMO	d_8_-17OHP-diMO	1.86/1.93	389.1	228.1/268.1/286.2	34/25/25	0.004
	Pregnanolone	PONE-MO	^13^C_2_-d_2_-Preg-MO	2.24	348.1	100.0	29	0.004
	Allopregnanolone	Allo-MO	d_5_-Allo-MO	2.12	348.2	100.0	29	0.003
Corticoids	Cortisone	E-diMO	d_4_-F-diMO	2.73/2.80	419.2	300.1/316.1/357.1	26/30/29	0.004
	Cortisol	F-diMO	d_4_-F-diMO	3.18/3.53	421.2	284.1/359.2	28/27	0.033
	Corticosterone	B-diMO	d_8_-B-diMO	2.65/3.07	405.1	343.1	28	0.004
	11-Deoxycortisol	S-diMO	d_4_-F-diMO	2.54/2.69	405.2	286.2/343.2	25/25	0.004
	11-Deoxycorticosterone	DOC-diMO	d_8_-B-diMO	1.79/2.03	389.1	126.0/138.0/327.2	40/40/28	0.004
	Aldosterone	A-diMO	d_4_-F-diMO	3.15/3.35	419.2	357.1	29	0.033
Internal standards	d_4_-estrone	d_4_-E1-MO	—	2.28	304.2	159.0/257.0	25/15	0.004
	d_2_-Androsterone	d_2_-AN-MO	—	2.28	322.3	257.1/290.21	18/18	0.004
	^13^C_3_-Testosterone	^13^C_3_-T-MO	—	2.25/2.47	321.3	129.2/141.1	29/30	0.004
	^13^C_2_-d_2_-Pregnenolone	^13^C_2_-d_2_-Preg-MO	—	2.19	350.3	304.3/104.3	21/27	0.004
	d_5_-Allopregnanolone	d_5_-Allo-MO	—	2.13	353.2	105.0	29	0.003
	d_9_-Progesterone	d_9_-P-diMO	—	0.91/1.12	382.3	292.2/333.3	28/28	0.032
	d_8_-17α-Hydroxyprogesterone	d_8_-17OHP-diMO	—	1.86/1.93	397.2	129.0/273.1/291.1	25/25/25	0.004
	d_4_-Cortisol	d_4_-F-diMO	—	3.18/3.53	425.2	288.0/363.0	28/29	0.033
	d_8_-Corticosterone	d_8_-B-diMO	—	2.65/3.08	413.3	349.3	29	0.004

T_r_: retention time; *D*_t_: dwell time. ^a^Retention time for one or two eluting peaks (from isomeric forms) per compound is reported. ^b^Number of product ions generated. ^c^Collision energy applied for generating each of the product ions.

Choice of stationary phase has a strong impact on selective separation of analytes on UPSFC^19,21^. Three different stationary phases (BEH, BEH 2-EP and CSH fluorophenyl (3.0 mm × 100 mm, 1.7 µm)), were used for initial screening of the steroids. The peak shape and resolution power of each steroid was evaluated. The mobile phase consisted of CO_2_ (A) and 0.1% formic acid in methanol-isopropanol (1:1) as co-solvent (B). The general screening gradient started with 2% B and linearly increased to 17% B in 2 min. Preliminary results indicated that BEH was the most promising stationary phase, since it provided the best peak shapes and resolution of the isomeric/isobaric pairs of steroids such as testosterone/dehydroepiandrosterone, androsterone/etiocholanolone, 17α-hydroxyprogesterone/11-deoxycorticosterone, and corticosterone/11-deoxycortisol. Therefore, the BEH column was selected for further method development.

The addition of additives (acid or base) at low concentration in the mobile phase increases the solubility of derivatised steroids and thereby results in more symmetric peak shapes^14^. Six co-solvent compositions with or without additives were investigated. Methanol and mixtures of methanol, isopropanol and/or acetonitrile were tested. Ammonium hydroxide and formic acid served as additives.

In the current study, separation of nineteen steroids together with their corresponding internal standards was successfully achieved on a BEH column within a few minutes. The retention of basic oxime derivative analytes on polar BEH stationary phase could be due to strong ionic interaction of free silanol groups available at the surface of this stationary phase^21^. For polar stationary phase like BEH, an increase in polarity should increase the retention time of analytes while molar volume has a reverse influence^22^. For example, cortisol which is more polar than cortisone elutes later and a similar pattern can be deduced for steroids of all four classes.

The co-solvent containing 0.1% formic acid in methanol-isopropanol (1:1) was selected as there was no advantage gained in using any of the other five co-solvents. However, in the process of mobile phase co-solvent B optimisation, the addition of the weak acid decreases the retention time of the analytes without any observable effect on the separation or peak shape of the steroid analytes^19^. Resolutions of the isomeric/isobaric analyte pairs were improved by selecting the column length of 150 mm. The flow rate, column temperature, back pressure, and make-up solvent conditions were optimised by additional tests. The optimised separation conditions have been described in the experimental section (see method section).

A slightly modified validation procedure as described in the EU Commission Decision/657/EEC was used as a guideline in this study. The process was performed by determining the linear range, accuracy, precision, limit of quantification (LOQ), and recovery (Table 2). Fresh standard solutions of the steroid analytes were used for all validation determinations. Matrix specific validation is often desired in steroid analysis owing to presence of differentinterfering components. Due to the presence of unknown amounts of endogenous steroids plasma cannot be used directly as a blank, steroids free sample. Therefore, different approaches have been employed to solve this issue such as use of artificial plasma, surrogate analyte, standard addition, background subtraction etc^23,24^. However, in our study the linear range of the method was determined from the calibration curves for each analyte in steroid-free plasma prepared by charcoal stripping (see method section). The square of the correlation coefficient (r^2^) was >0.998 for all the steroids (Table 2). It illustrates that the signal generated for each analyte was linear within the selected concentration. The linearity range obtained from this study was comparable to those already published SFC-MS/MS results^17,18^. The back-calculated concentration of the calibration samples was within ± 12% of the nominal value. No significant endogenous matrix interferences were observed and there were no noticeable co-eluting compounds in the plasma samples. Carry-over did not generate any problem, as all analytes were undetectable from blank injections after injecting the highest quality control calibrator.

**Table 2:** Method validation parameters.

No.	Compound	R^2^	Linear range (ng/mL)	LOQ (ng/mL)	Low concentration^a^		Medium concentration^a^		High concentration^a^		Absolute recovery (%)
					Accuracy (Bias)	Precision (CV%)	Accuracy (Bias)	Precision (CV%)	Accuracy (Bias)	Precision (CV%)	
1	E1	0.9993	0.05–30	0.05	−0.1	2.6	3.5	9.9	2.9	7.3	97.7
2	DHEA	0.9998	0.1–30	0.10	0.1	11.4	-2.7	14.8	4.8	4.3	95.8
3	AN	0.9999	0.05–30	0.05	8.3	4.2	5.0	7.6	8.0	0.5	94.7
4	ECN	0.9996	0.5–30	0.50	2.6	2.1	1.4	7.0	3.1	4.7	96.1
5	AE	0.9999	0.05–30	0.05	2.4	2.5	9.0	3.7	4.2	0.7	96.8
6	T	0.9998	0.05–30	0.05	5.6	6.1	15.6	10.2	12.2	12.2	87.1
7	DHT	0.9994	0.05–30	0.05	7.1	5.2	5.0	6.9	2.1	9.2	90.0
8	Preg	0.9986	0.1–30	0.10	2.9	3.1	11.8	3.6	0.3	4.3	94.4
19	17OHPreg	0.9985	0.5–30	0.5	10.1	4.3	3.1	1.7	3.5	6.1	88.3
10	P	0.9999	0.05–30	0.05	15.9	0.9	5.3	1.9	13.7	0.8	89.1
11	17OHP	0.9999	0.25–30	0.25	13.1	3.5	3.7	3.8	0.1	4.7	81.9
12	PONE	0.9995	0.05–30	0.05	2.4	1.7	17.5	4.8	21.6	9.5	91.3
13	Allo	0.9980	0.05–30	0.05	2.2	2.4	5.3	4.8	2.3	5.1	95.8
14	E	0.9999	0.05–500	0.05	9.2	2.6	18.8	1.7	9.3	2.8	102.8
15	F	0.9992	0.05–500	0.05	16.3	3.0	18.6	1.2	18.9	1.3	104.1
16	B	0.9991	0.05–500	0.05	11.0	2.4	20.1	3.7	12.7	2.5	102.3
17	S	0.9992	0.05–500	0.05	7.9	8.7	13.1	2.3	7.8	2.3	95.7
18	DOC	0.9983	0.05–250	0.05	9.3	6.4	8.0	5.4	8.9	7.1	82.2
19	A	0.9998	1–500	1.00	13.8	15.1	10.7	4.0	8.0	5.0	107.2

R^2^, correlation coefficient; LOQ, limit of quantification; CV%, coefficient of variance; E1, estrone; DHEA, dehydroepiandrosterone; AN, androsterone; ECN, etiocholanolone; AE, androstenedione; T, testosterone; DHT, dihydrotestosterone; Preg, pregnenolone; 17OHPreg, 17α-hydroxypregnenolone; P, progesterone; 17OHP, 17α-hydroxyprogesterone; PONE, pregnanolone; Allo, allopregnanolone; E, cortisone; F, cortisol; B, corticosterone; S, 11-deoxycortisol; DOC, 11-deoxycorticosterone; A, aldosterone. ^a^The low, medium and high concentrations for compounds 1–13 are 0.25, 2.5 and 20 ng/mL, respectively. The low, medium and high concentrations for compounds 14–19 are 2.5, 20 and 250 ng/mL, respectively.

The quantitative recovery of steroids in plasma was evaluated by comparing peak areas of the analytes in the reconstitution solvent with peak areas after extraction of steroids from plasma. Multiple aliquots (n = 6) at each of the three different concentrations were assessed. The mean recovery of steroids and corresponding deuterated internal standards ranged from 82–107%. Since we used corresponding deuterated internal standards and matrix matched calibration in plasma, the signal enhancement issue was taken into account.

Precision and accuracy were assessed by replicate analysis (n = 6) of spiked plasma samples at three different concentrations and data are presented in Table 2. The intraday and interday precisions were between 1% and 10% for most of the steroids and the accuracy was within ± 15% (Table 2). The lowest concentration levels that could be determined with a bias and CV% lower than ± 15% was considered as LOQ and found to be less than 0.1 ng/mL for most of the steroids, with some exceptions.

We have been able to apply the developed method in the KARMA study (Karolinska Mammography Project for Risk Prediction of Breast Cancer, KARMA) at Karolinska Institutet in Sweden, one of the world’s best-characterised breast cancer cohorts^25^, for diagnostic evaluation of steroidomics in plasma. The quality of the KARMA plasma samples has already been validated through proteomic profiling^26^. All blood samples were handled in accordance to a strict 30-hours cold-chain protocol and were processed in the high-throughput biobank at Karolinska Institutet. Information on risk factors and exposures were collected by questionnaire at study enrolment. Each study participant signed an informed consent form and accepted linkage to the national breast cancer register. The study was approved by the Stockholm ethical review board (2010/958–31/1). All experiments were performed in accordance with relevant guidelines and regulations.

We analysed all nineteen different steroids in plasma from more than 700 breast cancer patients and 1400 matched controls in the four major classes (estrogens, androgens, progestogens and corticosteroids) of steroids. The analytical time was 5.0 min per sample. The results will be reported separately. Furthermore, we have already published some more applications based on this method in peer-reviewed journals showing its applicability in biological samples^27,28^.

### Concluding remarks

This study focused on proving the use of UPSFC–MS/MS as an alternative method to LC–MS/MS and GC–MS/MS for the separation and quantification of endogenous steroids in human plasma. Whether UPSFC-MS/MS is truly a “green technology” in terms of its organic solvent consumption is still a matter of debate, but it definitely surpasses the LC-MS/MS and GC-MS/MS in terms of resolution and sensitivity. This UPSFC–MS/MS method is novel and provides simultaneous analysis of nineteen endogenous steroids from all four major classes within 5 min. Inclusion of a derivatisation step prior to analysis improved sensitivity of detection and outweighed the drawback of an increased sample preparation time. The validation data demonstrates that it is possible to identify and quantify these steroid analytes in small plasma sample volumes. Besides research applications and routine clinical screening, this method could be of specific interest in the analysis of steroids in biobanked samples where the availability of sample is generally limited. Therefore, the developed UPSFC–MS/MS method could be the method of choice for the diagnosis and monitoring of endocrine diseases due to its high throughput and sensitivity over immunoassays.

## Methods

### Materials

Estrone (E1), dehydroepiandrosterone (DHEA), androsterone (AN), etiocholanolone (ECN), testosterone (T), dihydrotestosterone (DHT), androstenedione (AE), pregnenolone (Preg), 17α-hydroxypregnenolone (17OHPreg), progesterone (P), 17α-hydroxyprogesterone (17OHP), pregnanolone (PONE), allopregnanolone (Allo), cortisone (E), cortisol (F), corticosterone (B), 11-deoxycortisol (S), 11-deoxycorticosterone (DOC), aldosterone (A) and the internal standards 2,4,16,16-d_4_-estrone (d_4_-E1), 16,16-d_2_-androsterone (d_2_-AN), 2,2,4,6,6,17α,21,21,21-d_9_-progesterone (d_9_-P), 2,2,4,6,6,21,21,21-d_8_-17α-hydroxyprogesterone (d_8_-17OHP), and 9,11,12,12-d_4_-cortisol (d_4_-F) were purchased from Steraloids Inc. (Newport, RI, USA). Methoxyamine hydrochloride, 2,3,4-^13^C_3_-testosterone (^13^C_3_-T), 20,21-^13^C_2_-16,16-d_2_-pregnenolone (^13^C_2_-d_2_-Preg), 2,2,3,4,4-d_5_-allopregnanolone (d_5_-Allo), and 9,11,12,12-d_4_-corticosterone (d_4_-B), highest purity solvents and chemicals were bought from Sigma-Aldrich (Stockholm, Sweden), unless otherwise stated. Water was distilled and deionised with a Milli-Q purification system (Millipore, Bedford, MA, USA). Human cohort plasma samples from healthy blood donors were obtained from the Academic Hospital, Uppsala, Sweden. Blood was collected from each participant by venepuncture into EDTA vacutainer tubes. The blood was centrifuged at 3500 g for 15 min; the plasma aliquoted and stored at −80 °C until further use. The plasma steroid levels are very stable for one year and special precautions to conserve the plasma were not required.

### Preparation of standard solutions and plasma free of steroids

Stock solutions of 1 mg/mL were prepared for all compounds using methanol and acetonitrile (1:1) as solvent, except for E1 (acetone) and 17OHPreg (methanol, acetonitrile, chloroform, 1:1:1). The steroids were assigned to two groups: group I included the estrogens, androgens and most of the progestogens, while group II consisted of the corticosteroids and 17OHP. A mixture of the nine internal standards (IS) was prepared in butylated hydroxytoluene (BHT, to prevent spontaneous oxidation) enriched methanol (0.05 mg/mL) at a concentration of 1 ng/mL for group I (d_4_-E1, d_2_-AS, ^13^C_3_-T, d_9_-P, ^13^C_2_-d_2_-Preg and d_5_-Allo); 5 ng/mL for d_8_-17OHP; and 100 ng/mL for group II (d_4_-F and d_4_-B). In order to have a matrix similar to the true samples, 400 mg of activated charcoal was added to 10 mL of normal human plasma to prepare plasma free of steroids as described by Aburuz *et al*.^29^. The prepared solutions and steroid-free plasma were stored at −80 °C until further analysis.

### Method validation

Analyte free human plasma samples were used for method validation after optimization of the method. Linearity, accuracy, precision, limit of detection (LOD), LOQ and recovery were determined for all nineteen steroids.

The calibration curves comprised of seven different concentrations (n = 4) of each analyte. The concentrations of the spiked steroid mix solutions were in the range of 0.05–30 ng/mL for group I, and 0.05–500 ng/mL for group II. These ranges were selected after consideration of clinically relevant concentrations for the steroids in normal plasma samples. Steroid-free plasma without spiking was selected as the blank. To each sample, 50 µL of the IS mixture was added. The double blank plasma sample was prepared without adding IS. The response i.e. the IS concentration multiplied by the peak area ratio (analyte/IS) of each steroid was plotted against the corresponding concentrations. The linearity was evaluated by using linear regression.

Accuracy and precision were calculated as bias (subtraction of the actual concentration from the measured concentration, reported as a percentage of the actual concentration) and coefficient of variance (CV%), respectively. Intraday accuracy and precision were determined by analysing six samples spiked at three different concentrations of both group I analytes (low, 0.25 ng/mL; medium, 2.5 ng/mL; high, 20 ng/mL) and group II analytes (low, 2.5 ng/mL; medium, 20 ng/mL; high, 250 ng/mL) on the same day. The interday accuracy and precision were calculated as the triplicate analysis of spiked samples at above concentrations on five consecutive days. The concentration in each sample was calculated using the calibration curve. The acceptable limits for both accuracy and precision should not exceed 20%.

LOD and LOQ was determined as the lowest concentration which provided a signal-to-noise ratio (S/N) greater than 3 and 10, respectively, by repeated injection (n = 6) with RSD of replicates below 15%.

Absolute recovery was assessed by comparison of the response i.e. the IS concentration times the peak area ratio (analyte/IS) of each steroid obtained after replicate analysis of standard solutions (n = 6) in solvent with the response of spiked analytes at three different levels ((low, medium and high) in steroid-free plasma. Matrix effect was assessed by comparing the peak area response of each steroid and IS from the post extraction spiked plasma and the peak area response of standard analyte solution at the same concentration (n = 6). The percentage of area difference indicates the ionisation behaviour of the analytes. If the observed value is greater or lower than 100% it indicates ionisation enhancement or suppression, respectively.

### Sample preparation

#### Extraction

Sample preparation commences with liquid-liquid extraction (LLE). A slightly modified method of steroid extraction was used^28^. Briefly, 50 µL of plasma was mixed with the mixture of IS. Plasma steroids were extracted to 2 mL of *tert*-butyl methyl ether (MTBE). Samples were gently vortexed for 10 min and were centrifuged at 1000 g for 10 min. The supernatant was collected and the solvent was evaporated under a stream of nitrogen gas. During the extraction, the steroids were protected against oxidation by the addition of 0.05 mg/mL BHT to the extraction solvent (MTBE). MTBE, diethyl ether, dichloromethane and a mixture of hexane and diethyl ether are the most common solvents used for LLE^7^. MTBE was found to be the best for a satisfactory extraction yield after testing a few solvents and solvent mixtures. Optimisation of the extraction procedure was controlled by thin layer chromatography as described in Ubhayasekera *et al*.^30^.

#### Derivatisation

Methoxyamine hydrochloride (20 mg/mL) in anhydrous pyridine was used as the derivatisation reagent. After addition of 100 µL of reagent, the samples were incubated at 60 °C for 45 min. The excess reagent was evaporated under a stream of nitrogen and oxime derivatives were dissolved in 50 µL of 0.1% formic acid in methanol-isopropanol (1:1). Samples were kept under −20 °C prior to analysis by UPSFC–MS/MS as described below.

### Determination of steroid concentrations by UPSFC–MS/MS

The analysis was performed by ultra-performance supercritical fluid chromatography–tandem mass spectrometry (UPSFC–MS/MS) on an Acquity UPC^2^ (Waters Corporation, Milford, USA) system coupled to a Xevo TQ-S triple quadrupole mass spectrometer (Waters, Milford, USA). The UPSFC system was equipped with a binary solvent delivery pump, an autosampler, a column oven, and a back pressure regulator. UPSFC was connected with the mass spectrometer by the commercial interface kit (Waters) composed of two T-pieces enabling the backpressure control and post column infusion with a make-up solvent.

The column selectivity was assessed by three different stationary phases (Acquity UPC^2^ columns (Waters, Milford, USA) BEH, BEH 2-EP and CSH fluoro-phenyl (3.0 mm × 100 mm, 1.7 µm)). Separation of the nineteen steroids was accomplished using an Acquity UPC^2^ BEH column. The column was kept at 40 °C and at a mobile phase flow rate of 2 mL/min. The gradient program started with 98% A (CO_2_) and 2% B (0.1% formic acid in methanol-isopropanol (1:1)), and maintained for 0.1 min, linearly increased to 17% B over 3 min, held at 17% B for 0.5 min, followed by a linear gradient down to 2% B over 0.5 min. Finally it was held for 1 min at 2% B for the elution of ionic liquids out of the instrument, resulting in a total separation time of 5 min. The back pressure was set to 1500 psi (103.4 bar) and the injection volume was 1.0 μL. Elution from the SFC system into the MS system was aided by a make-up solvent (0.1% formic acid in methanol) at a flow rate of 0.2 mL/min.

Mass spectrometric detection was performed using electrospray ionisation in the positive ionisation mode (ESI^+^) with a capillary voltage of 2.8 kV, cone voltage of 30 V, and source offset of 30 V. Nitrogen and argon (0.15 mL/min) served as the desolvation gas and the collision gas, respectively. Desolvation temperature was maintained at 500 °C, and source temperature was set to 150 °C. Desolvation gas flow and cone gas flow were maintained at a rate of 750 L/h and 150 L/h, respectively. The nebuliser gas flow was set to 7.0 bar (101.5 psi). Collision energy was varied to optimise product ion formation. The data acquisition range was set for *m/z* 100–600. Standard solutions of the steroids at 10 µg/mL were introduced to the source at 10 µL/min using IntelliStart™ in infusion mode. Mass spectra for each analyte were recorded in MS and MS/MS mode. The quantification was based on a multiple reaction monitoring (MRM) method and collision energy and scan dwell time were set according to Table 1. MS/MS conditions and the method were confirmed by individual analysis of the standard steroids (50 ng/mL). Data were acquired, analysed and processed with MassLynx ™4.1 software (Waters, Milford, USA). Quantification of steroids was performed using the corresponding internal standard.
